# P02.35. Methodology in integrative medicine research: challenges and solutions from a randomized clinical control trial using adapted yoga

**DOI:** 10.1186/1472-6882-12-S1-P91

**Published:** 2012-06-12

**Authors:** S Toise, S Sears, M Schoenfeld, M Blitzer, M Marieb, J Drury, M Slade, T Donohue

**Affiliations:** 1 The Hospital of Saint Raphael, New Haven, USA; 2 East Carolina University, Greenville, USA; 3 Yale School of Medicine, New Haven, USA; 4 Banner Health Cardiovascular Institute of North Colorado, Greeley, USA; 5 Yale University, Yale Occupational & Environmental Medicine, New Haven, USA

## Purpose

This randomized controlled clinical study evaluated the efficacy of adapted yoga (vs. usual care) to reduce psychosocial risks, which have been clinically shown to impact morbidity and mortality in implantable cardioverter defibrillator (ICD) recipients. The ICD collects and records real-time cardiac data, which were used in the study.

## Methods

Forty-six patients participated from a hospital in Connecticut. All participants were administered validated measures on psychosocial risk factors at weeks one and eight of the eight-week intervention. Patients in the intervention group participated in a weekly adapted yoga class for eighty minutes for eight weeks with assigned home practices. Clinical measures, including patients’ current and past medical health status and device usage, were collected three months prior to the study, during the study, and at a six-month follow-up.

## Results

Data revealed that the yoga group’s overall shock anxiety decreased while the control group’s increased, t(4.43, 36), p<0.0001 (total). The yoga group had less shock anxiety, t(2.86,36) p=0.007 (mean consequence), greater overall self-compassion, t(-2.84,37), p=0.007 (total), and greater mindfulness (equanimity) regarding emotions, t(-2.10,37) p=0.04 (mindfulness), than the control group. Exploratory analyses utilizing a linear model (R^2^=.98) of the observed anti-tachycardia pacing (ATP) events revealed that the expected number of ATP events in the intervention group was significantly lower than the control group. Additionally, the expected number of ATP events increased more rapidly as a function of the initial ATP events for the control group than for the intervention group.

## Conclusion

Our study demonstrated psychological benefits from a program of adapted yoga compared to usual care for ICD recipients. Marked improvements were reported in total shock anxiety, self-compassion, sense of equanimity, and decreased likelihood of ATPs. The data supports the continuation of research in mind-body interventions to better understand the role of complementary medicine to address ICD-specific psychosocial stress and its potential contributory role in cardiac outcomes.

